# Best Practices in Evolving Privacy Frameworks for Patient Age Data: Census Data Study

**DOI:** 10.2196/47248

**Published:** 2024-03-25

**Authors:** Colin Moffatt, Jonah Leshin

**Affiliations:** 1 Datavant San Francisco, CA United States

**Keywords:** census, date of birth, deidentification, HIPAA, Health Insurance Portability and Accountability Act, k-anonymity, patient privacy, policy, reidentification risk

## Abstract

**Background:**

Over the previous 4 decennial censuses, the population of the United States has grown older, with the proportion of individuals aged at least 90 years old in the 2010 census being more than 2 and a half times what it was in the 1980 census. This suggests that the threshold for constraining age introduced in the Safe Harbor method of the HIPAA (Health Insurance Portability and Accountability Act) in 1996 may be increased without exceeding the original levels of risk. This is desirable to maintain or even increase the utility of affected data sets without compromising privacy.

**Objective:**

In light of the upcoming release of 2020 census data, this study presents a straightforward recipe for updating age-constrained thresholds in the context of new census data and derives recommendations for new thresholds from the 2010 census.

**Methods:**

Using census data dating back to 1980, we used group size considerations to analyze the risk associated with various maximum age thresholds over time. We inferred the level of risk of the age cutoff of 90 years at the time of HIPAA’s inception in 1996 and used this as a baseline from which to recommend updated cutoffs.

**Results:**

The maximum age threshold may be increased by at least 2 years without exceeding the levels of risk conferred in HIPAA’s original recommendations. Moreover, in the presence of additional information that restricts the population in question to a known subgroup with increased longevity (for example, restricting to female patients), the threshold may be increased further.

**Conclusions:**

Increasing the maximum age threshold would enable the data user to gain more utility from the data without introducing risk beyond what was originally envisioned with the enactment of HIPAA. Going forward, a recurring update of such thresholds is advised, in line with the considerations detailed in the paper.

## Introduction

A person’s age is a singular piece of information. It is linked inextricably to an individual, incrementing ceaselessly throughout life and unable to be modified (despite some having tried [1]). Age is a fundamental piece of information that we routinely use to describe or categorize a person. Age values commonly occur in health data, either directly or more usually as implied by the patient’s date of birth (often aggregated by the year of birth). Thus, age is a useful piece of information when looking to match 2 records pertaining to the same individual [2], and, therefore, within an anonymized data set containing person-level information, the presence of an age value contributes to the risk of reidentification.

With microdata records, a rule of thumb is that the relative amount of reidentification risk that any value contributes is inversely proportional to the number of people in the population who share that value. For example, the sex value “male” is low risk as almost half the population shares that same value. This extends to a combination of values from several fields. The extreme case is where a person’s value (or combination of values) matches no other in the data set, making that person unique and, therefore, resulting in a disclosure risk that is very high. Any value or combination of values that is shared by too few people is high risk, and many of the methods developed and implemented to reduce this risk—for example, aggregation and constraining—effectively increase the size of the group who share the same combination of values and so reduce the rarity of value combinations.

When working with data sets with US patients, the US decennial census data contain group-level counts that can be incorporated into risk calculations. For example, one may use the census to look up the size of the population of all female patients aged 90 years and older (the census value will serve as an approximation due to shifts in population over time). Note that it is the absolute count of patients that is most relevant, rather than the proportion of a population subgroup relative to the full population.

Age values are not represented equally across the range of all ages. It is natural and expected for age cohorts to reduce in size as a function of age due to increased rates of mortality. While historical events such as epidemics of malign diseases can affect cohorts differently and birth rates over time along with emigration and immigration play a role in cohort population size, in general, population counts are decreasingly frequent in older people. Hence, there are, for example, fewer nonagenarians (individuals aged between 90 and 99 years) than there are octogenarians (aged between 80 and 89 years). Thus, especially in the population of very advanced age people, the reidentification risk contributed by age increases year-on-year as the number of individuals who share the same age reduces. To mitigate this within a data set where sensitive information is also present, it is sensible to constrain age values to some maximum. The HIPAA (Health Insurance Portability and Accountability Act) of 1996, in its Safe Harbor approach to the deidentification of data sets [3], gives a maximum age of 90 years to which all higher values should be lowered.

The risk of reidentification, or “disclosure risk,” is related to the amount of utility that a data set of personal information contains. As the risk is reduced, so is the value of the data set in terms of its usefulness. Aggregation or constraining, while reducing risk, will inherently reduce the granularity of information the data contain, since these methods effectively introduce error to the raw data values, and the quality of any statistical inference based upon the data will suffer as a result. Thus, there is a trade-off between reducing the disclosure risk of a data set and maintaining a level of utility that is sufficient to address questions of interest.

Enabling more granular year-of-age information is beneficial for studying health outcomes in the older populations. Through improved diets, better medical care, and generally healthier lifestyles, people are living longer. In addition to these environmental factors, a spike in birth rates following World War II into the early 1960s has also contributed to the high proportion of individuals aged over 60 years currently living in the United States [4]. With the growth of the older population in the United States and abroad [5], the development of efficacious drugs and treatments for an aging population is becoming increasingly important [6].

This prompts the question of whether the age threshold given by HIPAA is still appropriate. While 90 years of age may have been a good choice 2 and a half decades ago, is it still a good choice today, or could it be raised, and data set utility improved as a result? In consideration of the ways in which an age value contributes to the disclosure risk of a data set, we make the case that this threshold can be increased without reducing disclosure risk.

## Methods

### Overview

Data from the previous 4 decennial US population censuses were acquired [7] and investigated. As HIPAA was enacted in 1996, presumably, the age-constraining guidance of age of 90 years was based upon data from the most recent census, which was in 1990. At the time of writing, the most recent census is that of 2010, so that is used in support of updated guidance through comparison to the corresponding 1990 census counts.

Analyses used data from the census’s population “PCT12” tables that consist of population counts grouped by combinations of state, sex, and single year of age (at the census year). Using these counts, we can constrain subsets of these 3 variables while aggregating across others. For example, we can sum the population counts across all states for all ages of 92 years and older, grouping by sex (ie, one sum for male and another for female individuals).

To illustrate trends over time, we performed similar computations on data from the 1980 and 2000 censuses. Having obtained these counts, we then compared them across different census years and generated illustrative plots. Findings are presented in simple graphs along with distributions across the United States illustrated by choropleth maps.

All analysis was performed in the computational language R (R Foundation for Statistical Computing). Given the small data storage and compute costs and the public availability of the data, we were able to perform all the analysis on personal computers.

### Ethical Considerations

The exploration of reidentification risks associated with varying specificities of age information for older individuals carries certain ethical implications. Although the data under study were publicly available, using it in combination with proprietary data sets and record linkage technologies may elevate the risk of reidentification. The analysis herein was carried out in a technical environment that did not contain any data sets to which the census data could be linked.

The degree of privacy protection afforded to any proprietary data set must be assessed in the context of possible linkages to readily accessible data sets. In fact, HIPAA stipulates those linkages to “reasonably available information” be accounted for in statistical determinations of privacy risk [3].

This analysis does not require review from an institutional review board as per the US Department of Health & Human Services regulations for the protection of human participants (45 CFR 46.104(d)) [8]. In particular, this is “Research that only includes interactions involving…survey procedures…” and meets the criterion that “The information obtained is recorded by the investigator in such a manner that the identity of the human subjects cannot readily be ascertained, directly or through identifiers linked to the subjects.”

## Results

### Overall US Population and Sex Considerations

**Figure 1:**
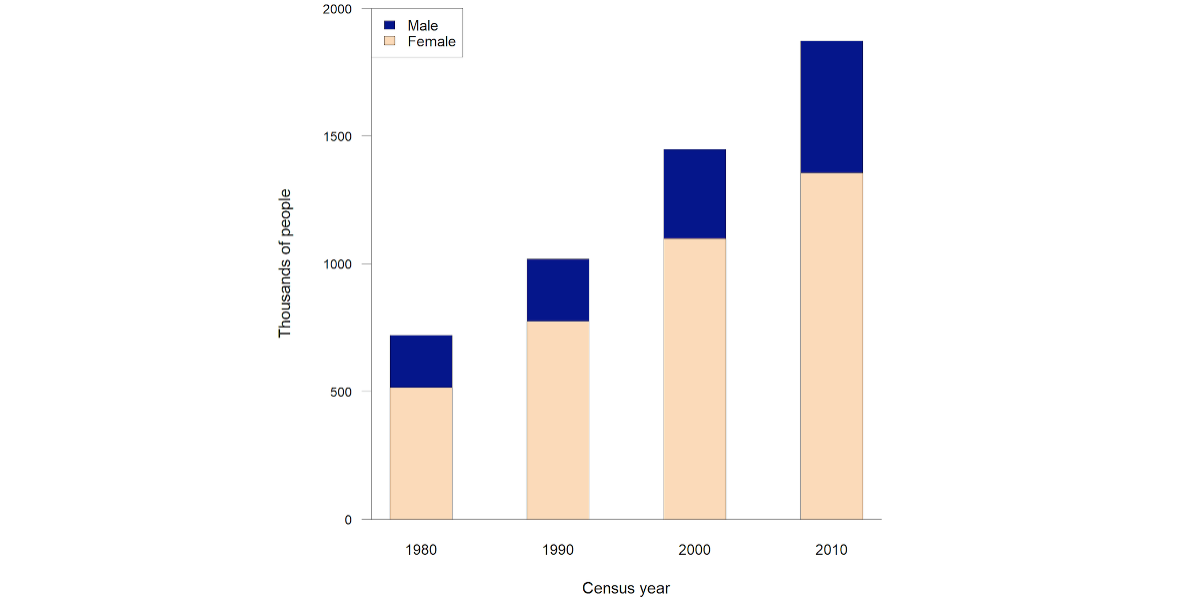
Male and female individuals (in 1000s) at least 90 years of age over the previous 4 decennial US population censuses.

The 1990 census showed that 1.02 million people were aged 90 years or older (Figure 1). In the 2010 census, this had risen by approximately 84% to 1.87 million. The increase was around 40% from 1990 to 2000 (a similar figure to the increase between 1980 and 1990) but smaller at 30% from 2000 to 2010. An increase was evident in both male and female individuals, although their increases were neither similar nor uniform.

**Figure 2:**
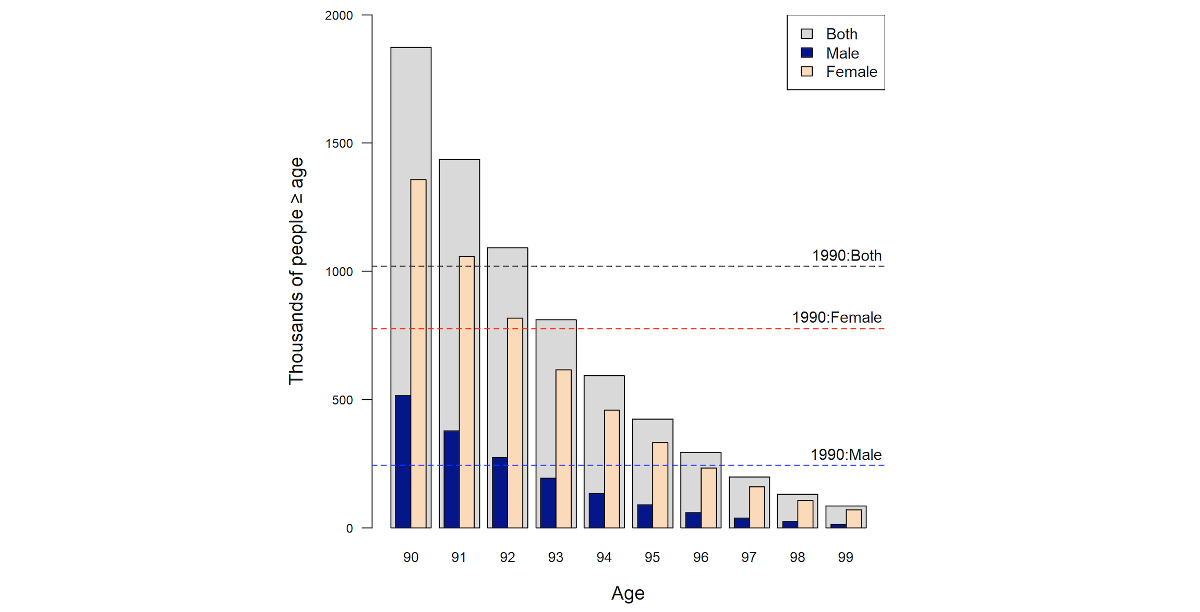
Male individuals, female individuals, and individuals of both sexes (in 1000s) at and above the given ages in the 2010 census. The dashed lines show the numbers of individuals who were aged 90 years and older in the 1990 census.

The figure of 1.02 million people who were aged 90 years or older was presumably the number that was deemed acceptable for HIPAA when it was enacted in 1996. In 2000, the census data began to include numbers for individuals aged between 90 and 99 years, so we could look up at which age, in 2010, the population was at least this size. With reference to Figure 2, we see that at the age of 92 years and above, the count in 2010 exceeded the required 1.02 million, but at the age of 93 years, it was below this number.

Since the numbers of male and female individuals at any age are far from equal (Figures 1 and 2), it can be argued that it is more sensible to consider the sexes separately, especially since sex values are normally present within a health data set. As there are fewer male than female individuals, presumably, the number of male individuals at least 90 years of age in the 1990 census (n=244,000; the blue dashed line in Figure 2) is a sufficiently large equivalence class size for either sex. In 2010, to achieve the same number, an age of 92 years would be required for male individuals, but for female individuals, it would be 95 years (since the number of people aged 96 years or older falls short by a few thousand).

### State Level Considerations

**Figure 3:**
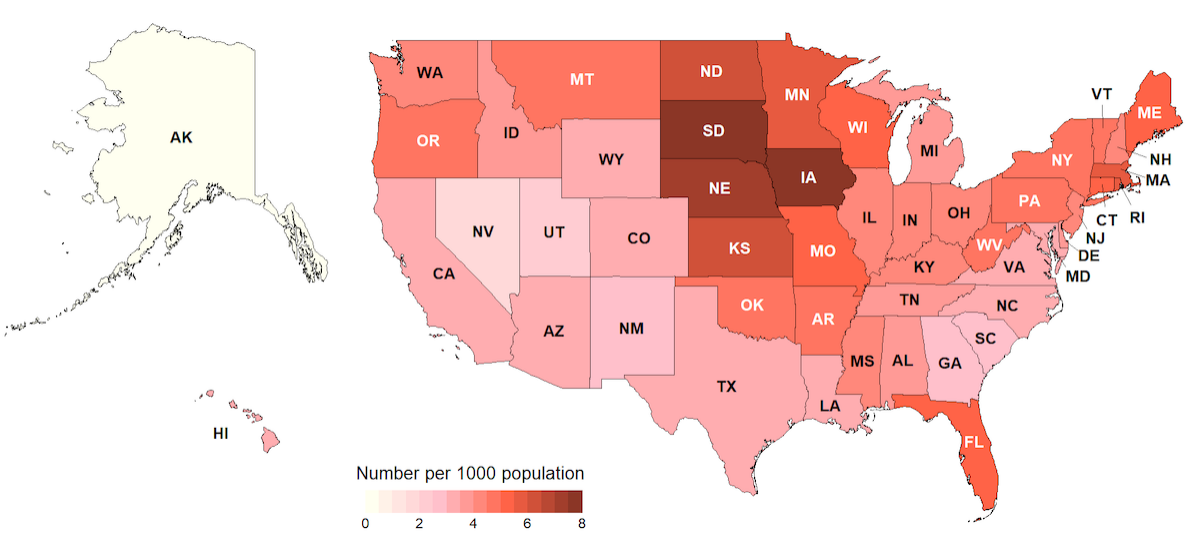
Choropleth map of the number of people per 1000 population who were aged 90 years or older in 1990 census by state. Alaska and Hawaii are shown (not to scale) to the left of the contiguous states.

**Figure 4:**
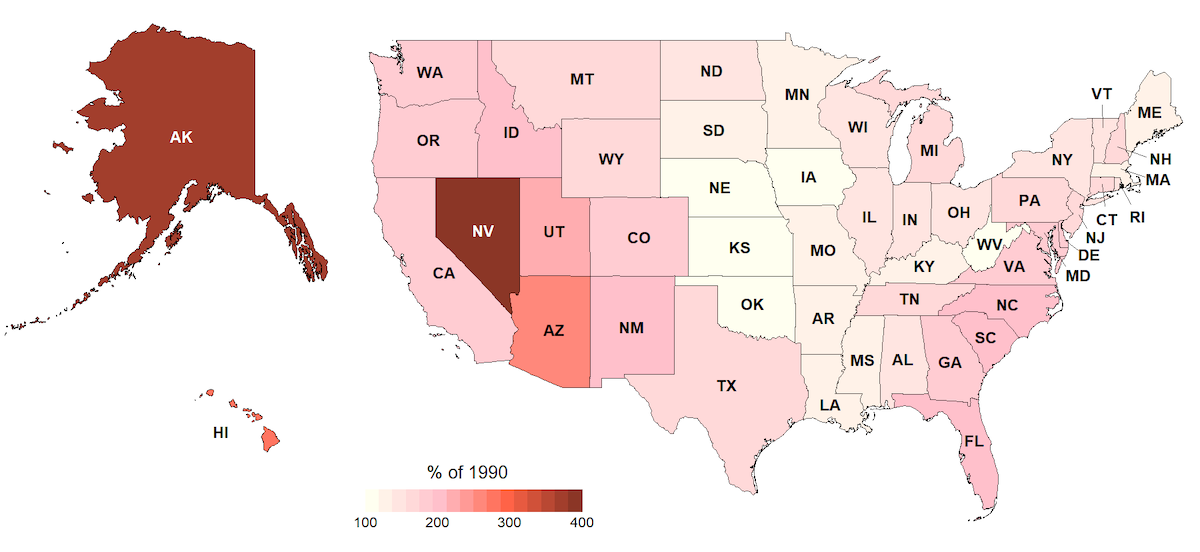
Choropleth map of the percentage change in the number of people aged 90 years or older between 1990 and 2010 by state. Alaska and Hawaii are shown (not to scale) to the left of the contiguous states.

A state-level examination of age is also worthwhile since state data are often included in health data sets. Results can best be illustrated by choropleth maps. Figure 3 shows shading based upon the proportion of people aged 90 years and older in the 1990 census. Alaska had the lowest proportion of people (0.7 per 1000) aged 90 years and older, followed by Nevada (1.8) and Utah (2.4), with the Midwest (Iowa=7.2) and Northeastern regions generally having higher values, along with Florida (4.9). Figure 4 shows the numbers of those aged 90 years or older in 2010 compared with 1990. Nevada has seen the greatest increase (411%), followed by Hawaii (402%), with all states having shown an increase of at least 134% (Nebraska) and those in the Midwest having generally seen a smaller increase.

With regard to absolute counts, among all states, Alaska had the smallest number of nonagenarians in 1990 with 358 (Wyoming was second with 1527). In 2010, although Alaska still had the smallest such population, the number increased by a factor of just over 4 to 1438 (Wyoming was second with 2899). If we assume that the decline in population by individual age year in Alaska follows a similar trend to that of the overall US population (Figure 2), it stands to reason that a maximum age cutoff of 93 years would maintain a level of anonymity at least equivalent to what the age-constraining threshold of 90 years achieved in 1990.

## Discussion

### Recommendations for the Present Day

The increase in longevity seen in the US population over recent decades means that constraining ages to 92 years would achieve at least as low a level of disclosure risk, based on the 2010 census, as 90 years did when it was introduced in HIPAA in 1996. This is desirable to implement as it effects an improvement in the utility of the data set if one accepts that keeping numbers “round” is an insufficient reason for continuing to use 90 years. Furthermore, if constraining is done differentially for the sexes, as is sensible if sex information is present in a data set, since female individuals tend to live longer, then female individuals’ ages can be constrained to 95 years, based on the 2010 census, and achieve the same level of risk protection as male individuals in 1990.

The increases in the proportions of the “oldest old” have not been uniform across the United States, with those underrepresented in 1990 going some way to “catch up” in 2010. HIPAA’s Safe Harbor method allows state values to be retained (along with 3-digit zip codes), so presumably constraining age to 90 years in 1990 was sufficient to have low enough risk levels in each state individually. Based upon the subsequent changes in Nevada and Alaska, there are strong grounds for raising the threshold to 93 years.

In addition to states, Safe Harbor also references 3-digit zip codes. This geographic unit consists of all zip codes with a fixed first 3 digits. Safe Harbor stipulates that 3-digit zips are permissible, provided that the area has a population greater than 20,000 according to the latest available census. It is worth noting that this threshold is based on the dynamic count of current census data rather than an underlying list of static 3-digit zips that are not permitted. This approach is consistent with our recommendation of basing age constraints on current population counts rather than on a static age threshold.

Our recommendation for raising the age-constraining threshold above 90 years is ultimately predicated on the assumption that population counts that combine age with other variables permitted by Safe Harbor would be at least as great as they were at the time HIPAA was enacted. For example, in 3-digit zips with populations above 20,000, we would assume that the count of individuals aged 93 years exceeds the corresponding count from 1990.

### Future State

With the release of a new census every 10 years, one may perform an analysis of this sort and provide updated recommendations. It is important, however, to consider the implications of making such updates over time. One consequence of decennial updates is that a reader of the data set must be aware of the age threshold that was used in order to properly analyze the data. Even as age thresholds are likely to increase over time, some health data sources will be late to adopt due to operational constraints. However, in the event that the age threshold decreased, data sources would be required to modify their age-binning procedures.

The emergence of privacy-preserving technology has enabled aggregate-level counts to be computed with statistical privacy guarantees [9,10]. When using counts that were computed through a privacy-preserving method that may alter the true count value, one must understand the quantitative framework used in the computation and modify the methodology accordingly. For example, when using a population count to derive an age-constrained value, one might use the lower end of a 1-sided 99% CI associated with the purported population count.

Due to these technology considerations, special care will be needed when using population counts from the 2020 census. In 2020, population counts were computed using differential privacy [11], which introduces an element of variance into the results. In larger geographic regions, the reported population count of a group with a given combination of variables such as age, sex, race, and ethnicity will have a smaller error relative to the true population size. In a smaller region, however, this variance could prove to be the difference between a group size that is large enough to protect anonymity to a sufficiently high standard and one that is not. Of course, a benefit of this variance is that it mitigates the reidentification risk that would result from revealing the true count of a small set of individuals with a rare combination of these variables.

The COVID-19 pandemic also impacted the 2020 census in several ways. First, the pandemic tragically took the lives of around 200,000 disproportionately older individuals [12] during the time of data collection, impacting demographic counts. The pandemic also impacted data collection operations due to unprecedented public health protocols and a politically charged environment, both of which resulted in undercounts of the true population [13,14].

### Further Considerations

It is routine for personal identifiable information to be replaced by a pseudorandom key that serves as a patient ID, enabling data sets to be linked without exposing an individual’s personal identifiable information. Such linking facilitates the creation of longitudinal data sets, which introduce additional considerations into privacy risk assessment.

For certain fields of a transient nature, such as state of residence or disease diagnosis, the presence of new values over time may enable an attacker to more effectively triangulate a patient’s information, elevating the risk of reidentification. A similar phenomenon can occur with a maximum age cutoff: if a data set from 5 years ago shows an individual to be 89 years old, yet a current data set shows the same individual (as identified by the same “linking token”) to be 90 years old, it is simple enough to deduce that they are actually 94 years old but have been constrained to 90 years.

Another consideration is that changes to the threshold pose a challenge for naive patient matching strategies that may use age or year of birth value as a component of a patient ID used to match patients across data sets (for example, using year of birth in combination with a hash of first name, last name, and address). It is advisable to instead include a patient’s actual (nonbinned) year of birth as part of the hashing input.

In practice, the only instance where current census data are referenced in HIPAA’s Safe Harbor criteria is the list of regions determined by the first 3 digits of a zip code. Any such region with a population under 20,000 is deemed to pose an unacceptable reidentification risk and is not permitted to be part of a Safe Harbor data set [15]. Any change to the Safe Harbor criteria, such as a dynamic age-constraining threshold, would need to come from the US Department of Health and Human Services.

From a regulatory perspective, expert determination [16] is the alternative approach to Safe Harbor for establishing that a data set is sufficiently deidentified by HIPAA standards. While Safe Harbor mandates a list of prescribed operations to be applied to a data set, expert determination allows a privacy expert to use discretion in the operations that must be performed on the data to render the risk of reidentification to be very small. Therefore, the expert determination method offers a readily available avenue for implementing these suggested new age thresholds.

Since expert determination allows for discretion, one can often make strategic modifications to a data set in order to retain certain fields at a desired level of granularity. For example, suppose that a data recipient was interested in keeping age values up to the age of 92 years. It may be the case that for individuals above the age of 90 years, the combination of other variables in the data set confers an unacceptable level of risk (note that in theory there could also be elevated levels of risk for individuals below the existing Safe Harbor age cutoff of 90 years). If the data contained 3-digit zip codes, an example of a possible mitigation would be to merge neighboring zips to reduce the risk of reidentification.

### Limitations

A caveat to these findings is the potential unreliability of the accuracy of the ages of such older people [3], as has been discussed particularly for centenarians [17]. This is due to a variety of causes, but we can expect to see improved reliability as time goes on.

In addition to questions of accuracy on the level of individual age reporting, there is a question of consistency across groups. Our findings consider age on its own and in the context of common sex and state variables. One variable we have not analyzed is ethnicity. Life expectancies are known to differ across ethnicities; moreover, the US census is known to have differing census reporting rates across ethnicities [18], which is influenced by a range of sociopolitical factors.

### Conclusion

Age is a critical variable when it comes to health data. While having a maximum age threshold is valuable for protecting patient privacy, such a threshold limits the granularity of insights into patient outcomes for the older population. Therefore, the threshold should be increased to the extent that it is possible to do so while staying below HIPAA’s original level of permitted risk.

The decennial release of updated demographic statistics is a sensible time to reevaluate accepted thresholds for variable constraints such as age. When using the census as a reference, thoughtful consideration must be given to both biases in the data collection as well as the use of privacy-preserving technologies.

## Abbreviations

HIPAA: Health Insurance Portability and Accountability Act

## Data Availability

The data sets generated during and/or analyzed during this study are available from the US Census Bureau repository [7].
